# Quantitative trait loci associated with amino acid concentration and *in vitro* protein digestibility in pea (*Pisum sativum* L.)

**DOI:** 10.3389/fpls.2023.1083086

**Published:** 2023-03-10

**Authors:** Junsheng Zhou, Zhongyang Wan, Krishna Kishore Gali, Ambuj Bhushan Jha, Michael T. Nickerson, James D. House, Bunyamin Tar’an, Thomas D. Warkentin

**Affiliations:** ^1^ Crop Development Centre, Department of Plant Sciences, University of Saskatchewan, Saskatoon, SK, Canada; ^2^ Department of Food and Human Nutritional Sciences, University of Manitoba, Winnipeg, MB, Canada; ^3^ Department of Food and Bioproduct Science, University of Saskatchewan, Saskatoon, SK, Canada; ^4^ Richardson Centre for Food Technology and Research, University of Manitoba, Winnipeg, MB, Canada

**Keywords:** pea, methionine, cysteine, tryptophan, lysine, protein digestibility, QTLs

## Abstract

With the expanding interest in plant-based proteins in the food industry, increasing emphasis is being placed on breeding for protein concentration and quality. Two protein quality traits i.e., amino acid profile and protein digestibility, were assessed in replicated, multi-location field trials from 2019 to 2021 in pea recombinant inbred line population PR-25. This RIL population was targeted specifically for the research of protein related traits and its parents, CDC Amarillo and CDC Limerick, had distinct variations in the concentration of several amino acids. Amino acid profile was determined using near infrared reflectance analysis, and protein digestibility was through an *in vitro* method. Several essential amino acids were selected for QTL analysis, including lysine, one of the most abundant essential amino acids in pea, and methionine, cysteine, and tryptophan, the limiting amino acids in pea. Based on phenotypic data of amino acid profiles and *in vitro* protein digestibility of PR-25 harvested in seven location-years, three QTLs were associated with methionine + cysteine concentration, among which, one was located on chromosome 2 (R^2^ = 17%, indicates this QTL explained 17% phenotypic variation of methionine + cysteine concentration within PR-25), and two were located on chromosome 5 (R^2^ = 11% and 16%). Four QTLs were associated with tryptophan concentration and are located on chromosome 1 (R^2^ = 9%), chromosome 3 (R^2^ = 9%), and chromosome 5 (R^2^ = 8% and 13%). Three QTLs were associated with lysine concentration, among which, one was located on chromosome 3 (R^2^ = 10%), the other two were located on chromosome 4 (R^2^ = 15% and 21%). Two QTLs were associated with *in vitro* protein digestibility, one each located on chromosomes 1 (R^2^ = 11%) and 2 (R^2^ = 10%). QTLs associated with *in vitro* protein digestibility, and methionine + cysteine concentration on chromosome 2 were identified to be co-localized with known QTL for total seed protein concentration in PR-25. QTLs associated with tryptophan and methionine + cysteine concentration co-localized on chromosome 5. The identification of QTLs associated with pea seed quality is an important step towards marker-assisted selection of breeding lines with improved nutritional quality, which will further boost the competitiveness of pea in plant-based protein markets.

## Introduction

Despite the currently relatively high sales price of the plant-based protein products, their popularity is increasing in recent years (Van Loo et al., 2020; Estell et al., 2021). Trends toward sustainable agriculture, healthy diets, food security, expanding global population, animal welfare, and religious beliefs are contributing to the expansion of plant-based protein product launches to meet the diverse needs (Sabate & Soret, 2014; Alcorta et al., 2021; Aschemann-Witzel et al., 2021).

Pea protein, as one of the common ingredients in the fabrication of meat alternatives, is rich in lysine, but relatively low in tryptophan and sulfur containing amino acids, such as methionine and cysteine (Leterme et al., 1990; Pilorge et al., 2021). To provide a complete and balanced set of amino acids, food companies need to complement pea protein with cereal protein, since cereals tend to be rich in the sulfur amino acids, but limiting in lysine (Srivastava et al., 2015). Compared to soybean protein, which is also widely used in plant-based protein products, pea protein is less allergenic, and contains less offensive off-flavors, such as beany, chalky flavors, and hence require less processing (Hoffmann et al., 2017; Taylor et al., 2021). These advantages could contribute to the wider use of pea protein in the future, but the current limitation of sulfur amino acids could also be an obstacle to wider use. Until now, most of the emphasis related to pea breeding for protein has been related to protein quantity instead of quality. But as the demands of the plant-based protein industries is continuously growing, a more profound study of pea protein quality traits is necessary.

Protein digestibility corrected amino acid score (PDCAAS) is an index that has been widely adopted when evaluating the quality of a specific protein. PDCAAS is based on both the amino acids profile and the digestibility of the proteins (Schaafsma, 2005). Amino acid profile is typically assessed by HPLC analysis, (Dołowy & Pyka, 2014; Sharma et al., 2014). HPLC analysis produces accurate results but is expensive and low throughput. Amino acids have distinct attributes, which increases the complexity of enzyme digestion steps. Three different digestion steps are required in HPLC analysis to assess the complete amino acid profile, and long incubation periods makes it a cumbersome method for amino acid profiling on large numbers of samples typical of plant breeding programs (Kambhampati et al., 2019). The use of near-infrared spectroscopy (NIR) has been widely used to assess the protein concentration in different crops. Recently, this non-destructive approach has been extended to assess the amino acid profile of legume seed samples (Hang et al., 2022; Shi et al., 2022). Shi et al. (2022) developed a calibration curve to predict the concentration of individual amino acids in soybean based on the NIR scans. The development and enhancement of the curve relies on data derived from established and validated wet chemistry reference methods, including the use of liquid chromatographic methods. For most amino acids, relatively high correlations were detected between the reference chemistry method, namely HPLC, and the NIR analysis. The correlation coefficients fell within a range of 0.76 to 0.91. Even for the least abundant amino acids, for instance, methionine, the correlation coefficient was 0.77. The benefits of using NIR relate to high throughput, low cost of analysis, and the potential to use intact seeds for non-destructive analysis (Jiang, 2020). The latter is particularly important when dealing with precious seed quantities such as those derived from breeding programs.

Protein digestibility reflects the extent to which a protein is broken down into its constituent amino acids and the latter made available for absorption within the gastrointestinal tract. *In vivo* and *in vitro* methods have been used to measure protein digestibility. The *in vivo* method assesses true fecal digestion in a rodent model and is accurate, but it has the limitations of high cost and low throughput, due to the significant involvement of animals in the analysis (Tavano et al., 2016). In contrast, *in vitro* methods simulate conditions within the digestive tract, including enzyme digestion, with protein digestibility assessed by various methods, including the change of pH of samples over a defined time course. This reproducible approach provides a method with relatively high throughput compared to the *in vivo* method and has been adopted to assess digestibility of different plant proteins (Duodu et al., 2002; Bessada et al., 2019; Ketnawa & Ogawa, 2021).

With more diverse needs from both consumers and the food industry, it has become necessary to develop crop varieties with improved protein quality. Understanding the underlying genetic control of protein quality traits is critical to aid in breeding such varieties. In the current study, we used PR-25, a recombinant inbred line population, to understand the genetic basis of protein quality traits in pea. PR-25 is derived from the cross of a medium protein parent CDC Amarillo and a high protein parent CDC Limerick. These two genotypes differed in individual amino acid concentrations from 10% to 27%, which makes PR-25 as a valuable source for the identification of QTL regions associated with protein quality traits. These QTL regions have the potential to be developed into breeder-friendly DNA markers, which could be used to improve the protein quality of pea and hence, increase the competitiveness of pea in plant-based protein markets.

## Materials and methods

### PR-25 mapping population

The PR-25 mapping population used in this study is a recombinant inbred line (RIL) population with 110 lines derived from the cross of CDC Amarillo x CDC Limerick. The mapping population was grown in seven locations during 2019 to 2021 in microplots (1 m^2^) and the harvested seed from individual plots was used in the current study. Information about PR-25, including its seeding date, harvest date and crop management in all locations were detailed in Zhou et al. (2022). Briefly, PR-25 was grown at Sutherland nursery in 2019 with 2 biological replicates. It was grown at Sutherland, Rosthern and Lucky Lake nurseries in 2020 and Floral, Rosthern and Lucky Lake nurseries in 2021 with 3 biological replicates per nursery. All nurseries were in Saskatchewan, Canada and among which, Sutherland and Floral are located in the Dark Brown soil zone, Rosthern is located in the Black soil zone, and Lucky Lake is located in the Brown soil zone. Best management practices for field pea production in western Canada were used at each location.

### Amino acid profiling and NIR calibration development

A total of 2359 whole seed samples, including 159 samples from 2017 GWAS (Gali et al., 2019) and 2200 samples from 2019-2021 PR-25, were used for calibration development and amino acid profile assessment (**Table 1**). They were stored at -20°C before any analysis. All samples were scanned in Dr. James House’s lab (University of Manitoba, Winnipeg, MB, Canada) *via* NIR spectroscopy on a PerkinElmer DA 7250 diode array NIR system (PerkinElmer Health Sciences Canada Inc.) to obtain spectral data. A sub-sample of 339, including all 159 samples from 2017 GWAS and 180 samples from 2019-2021 PR-25, were selected from 2359 samples for development and improvement of the calibration formula. These sub-samples were analyzed *via* HPLC for amino acid concentration and the protocol of HPLC was detailed by Shi et al. (2022). A prediction model was created using the whole seed spectrum and chemical data of 2017 GWAS samples, the calibration was then applied to all remaining samples to estimate chemical compositions (Shi et al., 2022). Annual maintenance and improvement of prediction model were made based on the HPLC data of selected samples from PR-25. Sixty samples each were chosen from year 2019 to 2021 to improve the accuracy of calibration formula. Samples in each year were divided into quartiles of predicted crude protein content in descending order and within each quartile, 15 samples were drawn randomly.

**Table 1:** Information of whole seed pea samples used for near infrared reflectance (NIR) calibration development and amino acid profile assessment.

Year	Population	Location	Lines	Replicates	Total number of tested samples	Use
**2017**	GWAS	Sutherland	80	2	159 (1 missing)	Calibration development
**2019**	PR-25	Sutherland	110	2	220	Calibration improvement/ Amino acid profiling
**2020**	PR-25	Sutherland	110	3	330	
	PR-25	Rosthern	110	3	330	
	PR-25	Lucky Lake	110	3	330	
**2021**	PR-25	Floral	110	3	330	
	PR-25	Rosthern	110	3	330	
	PR-25	Lucky Lake	110	3	330	
			**Total**		2359	

All seed samples were stored in a 4°C walk-in cooler prior to NIR scanning to avoid protein denaturation. After scanning with NIRS, approximately 20 grams of each selected sample was ground with a Retsch ZM-200 grinder (Retsch, Haan, Germany) using a 0.75mm sieve and then stored at -20 °C before further analysis.

Detailed information about NIR analysis of whole seeds, as well as wet chemistry analysis of protein and amino acid concentrations was reported by Shi (2021). Detailed information about the development of an NIR calibration model for pea amino acid concentration was reported by Hang et al. (2022).

### *In vitro* protein digestibility assessment

Sample preparations were conducted in the Grain Innovation Lab (Crop Development Centre, University of Saskatchewan). Five grams of pea seeds per line from 2019 PR-25 yield trials were ground into homogeneous powder using a cyclone sample mill model 3010-030 (UDY Corporation, USA). Protein digestibility was evaluated using an *in vitro* method. Detailed protocol of *in vitro* protein digestibility (IVPD) determination was described by Cabuk et al. (2018).

### Measurement of *in vitro* protein digestibility-corrected amino acid score

*In vitro* PDCAAS was calculated as follows based on Nosworthy et al. (2017):


$$\text{AAS}=\frac{\text{mg of most limiting amino acid in }1\text{g of tested protein}}{\text{mg of this particular amino acid in }1\text{g of reference protein}}$$

in vitro PDCAAS=AAS*IVPD

### Statistical analysis

**Figure 1:**
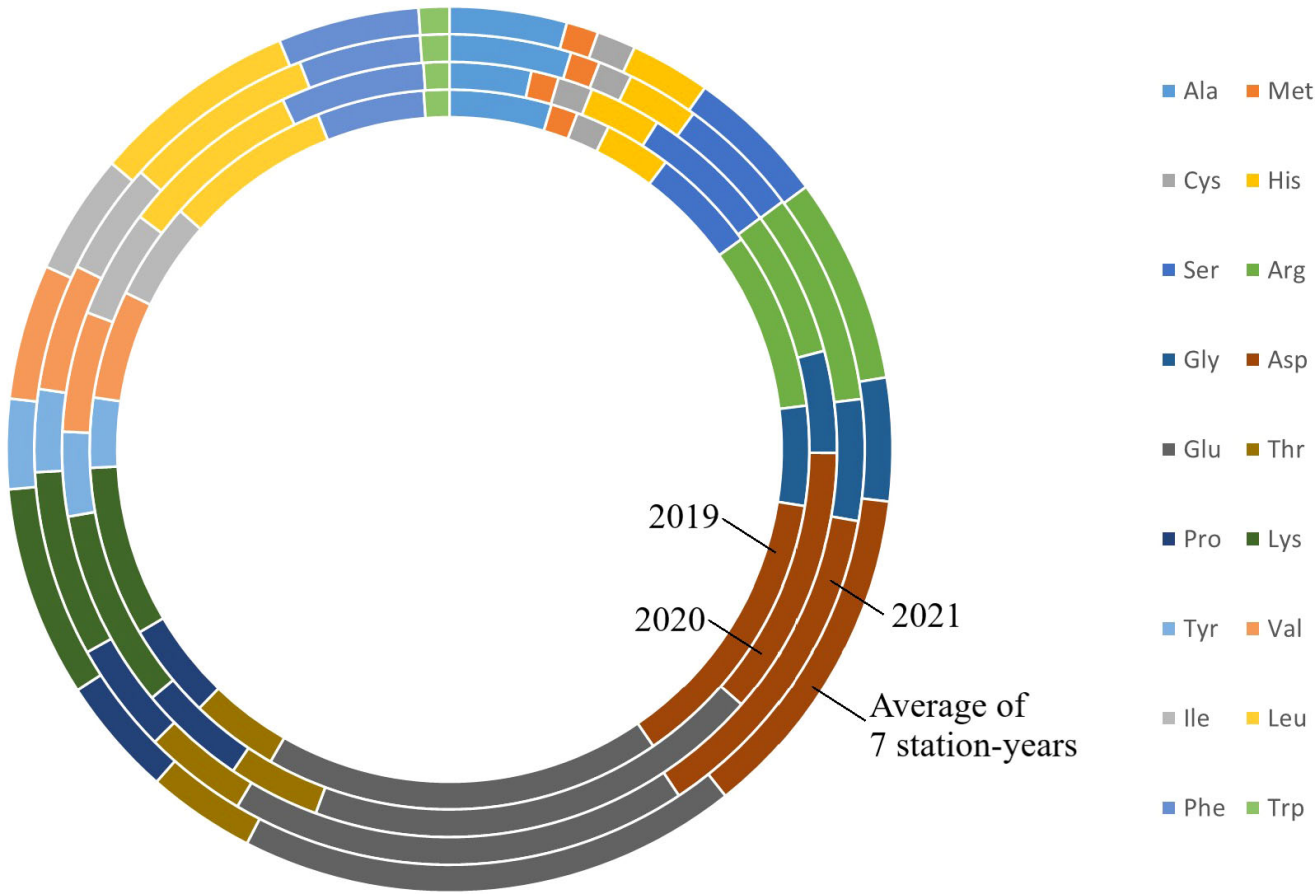
Amino acids profile of PR-25 from field trials conducted in Sutherland nursery in 2019, Sutherland, Rosthern, Lucky Lake nurseries in 2020 and Floral, Rosthern and Lucky Lake nurseries in 2021, and the average amino acid profiles of PR-25 across the seven station-years.

**Figure 2:**
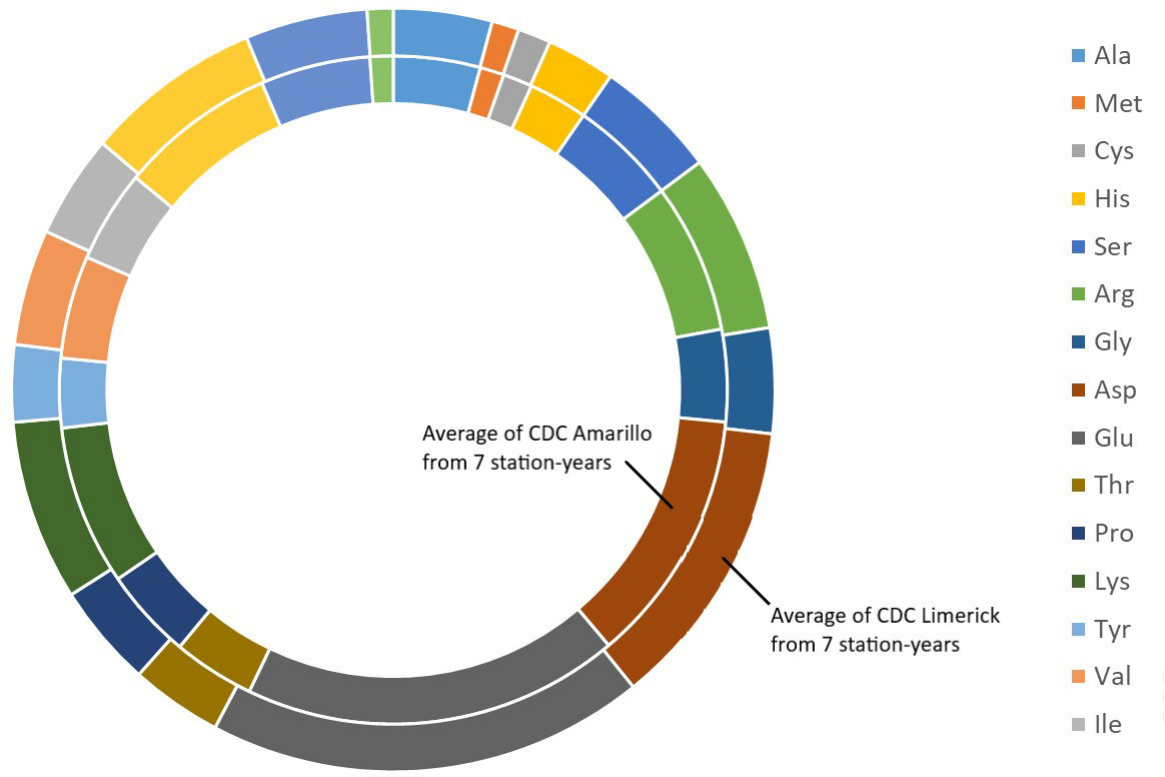
Average amino acids profile of CDC Amarillo and CDC Limerick from 7 station-years.

**Figure 3:**
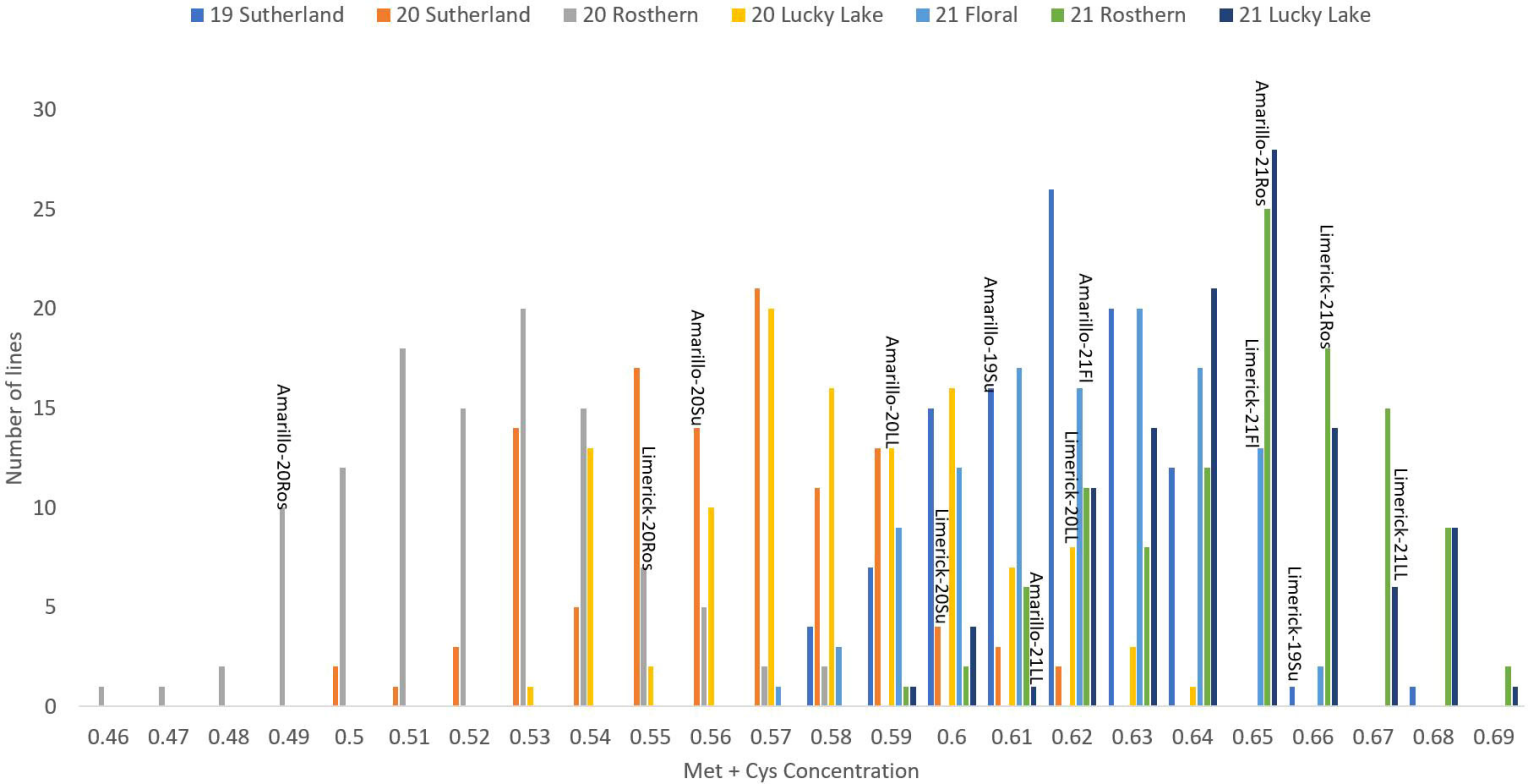
Frequency distribution of methionine + cysteine concentration of PR-25 across seven station-years based on the average of biological replicates for each line in each station-year (two biological replicates in 2019 Sutherland and three biological replicates for the rest of the station-years), and the averaged methionine + cysteine concentration for the parents of PR-25 in each station-year.

**Figure 4:**
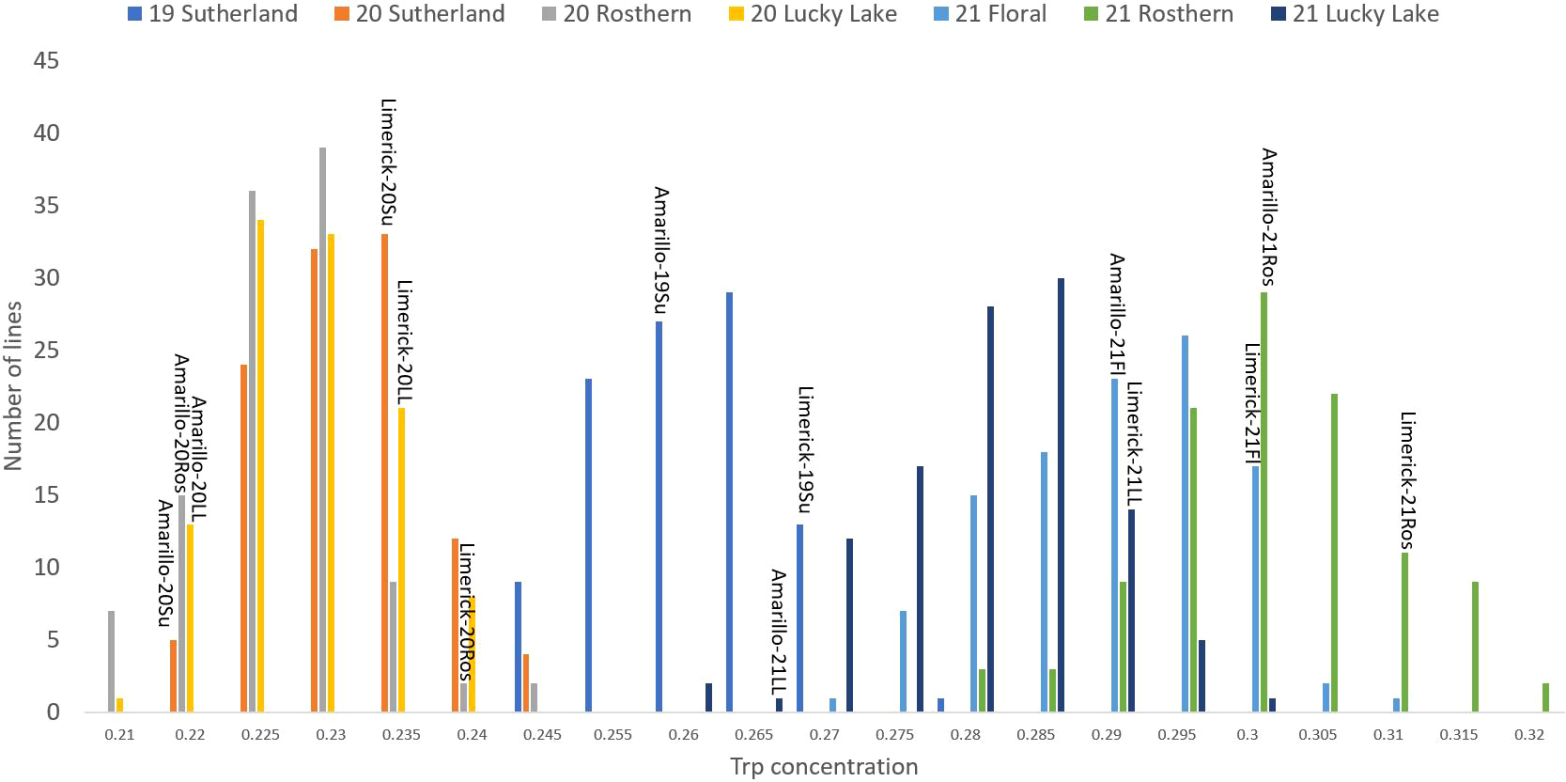
Frequency distribution of tryptophan concentration of PR-25 across seven station-years based on the average of biological replicates for each line in each station-year (two biological replicates in 2019 Sutherland and three biological replicates for the rest of the station-years), and the averaged tryptophan concentration for the parents of PR-25 in each station-year.

**Figure 5:**
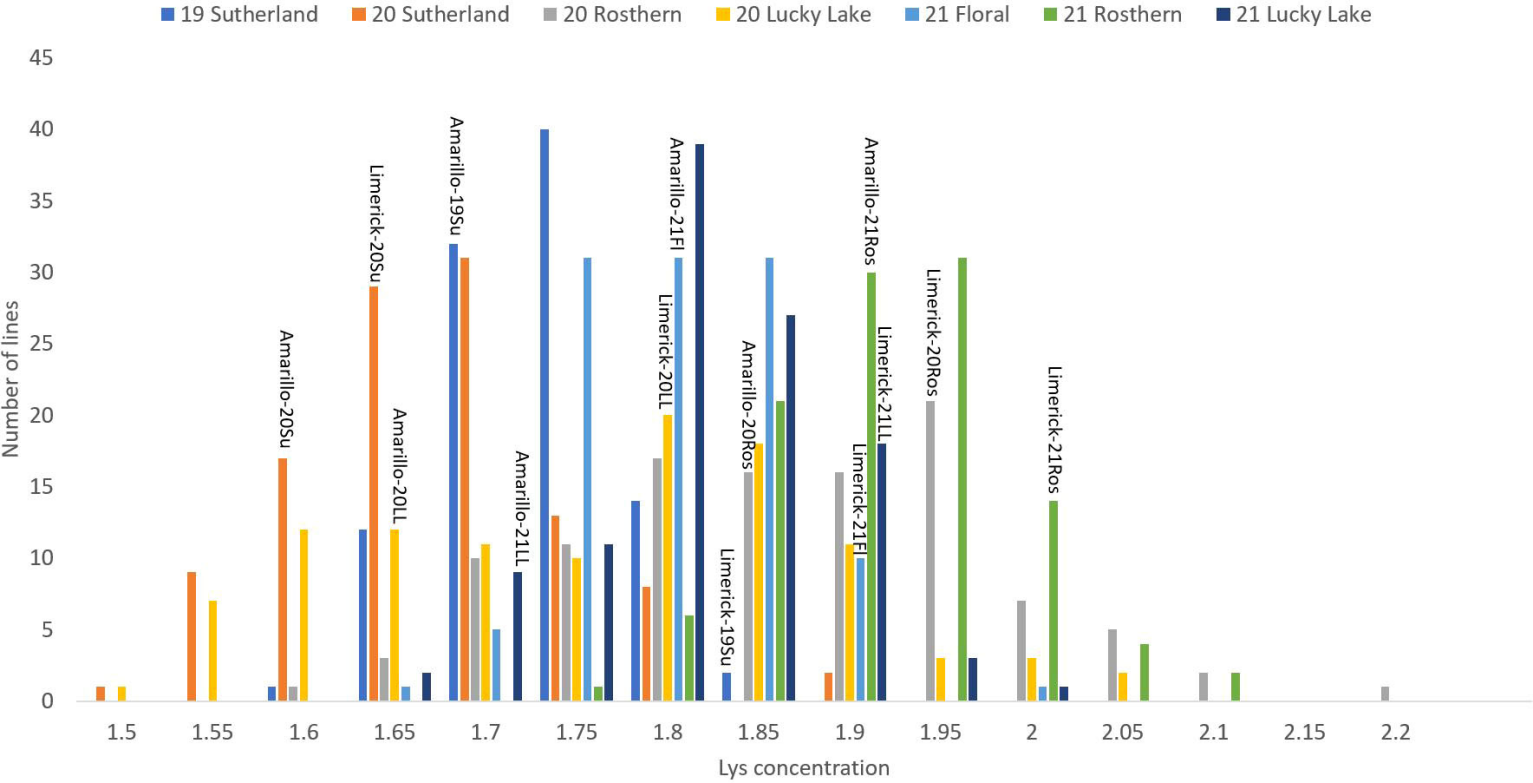
Frequency distribution of lysine concentration of PR-25 across seven station-years based on the average of biological replicates for each line in each station-year (two biological replicates in 2019 Sutherland and three biological replicates for the rest of the station-years), and the averaged lysine concentration for the parents of PR-25 in each station-year.

**Figure 6:**
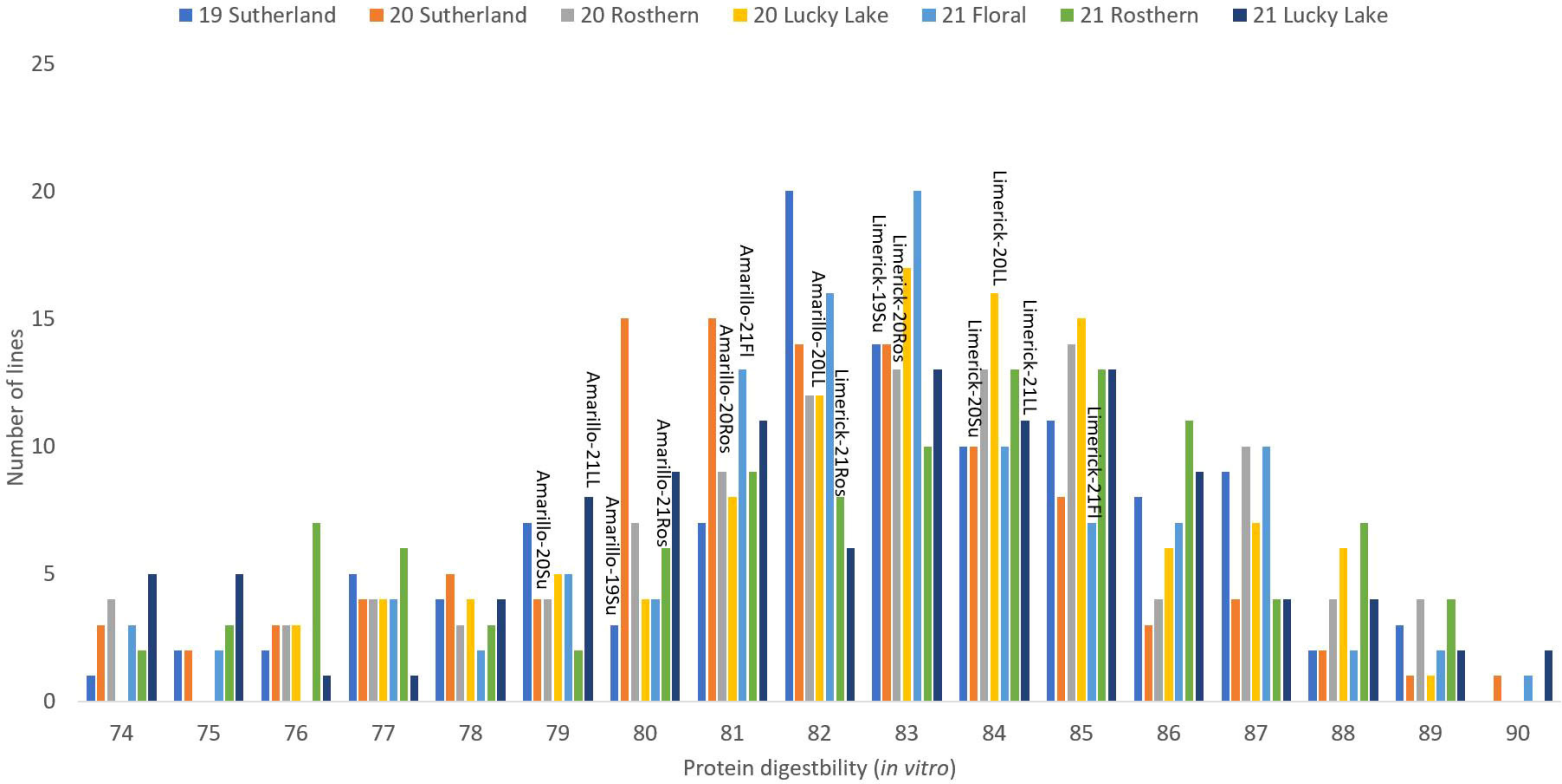
Frequency distribution of protein digestibility (*in vitro*) of PR-25 across seven station-years based on the average of biological replicates for each line in each station-year (two biological replicates in 2019 Sutherland and three biological replicates for the rest of the station-years), and the averaged protein digestibility (*in vitro*) for the parents of PR-25 in each station-year.

**Figure 7:**
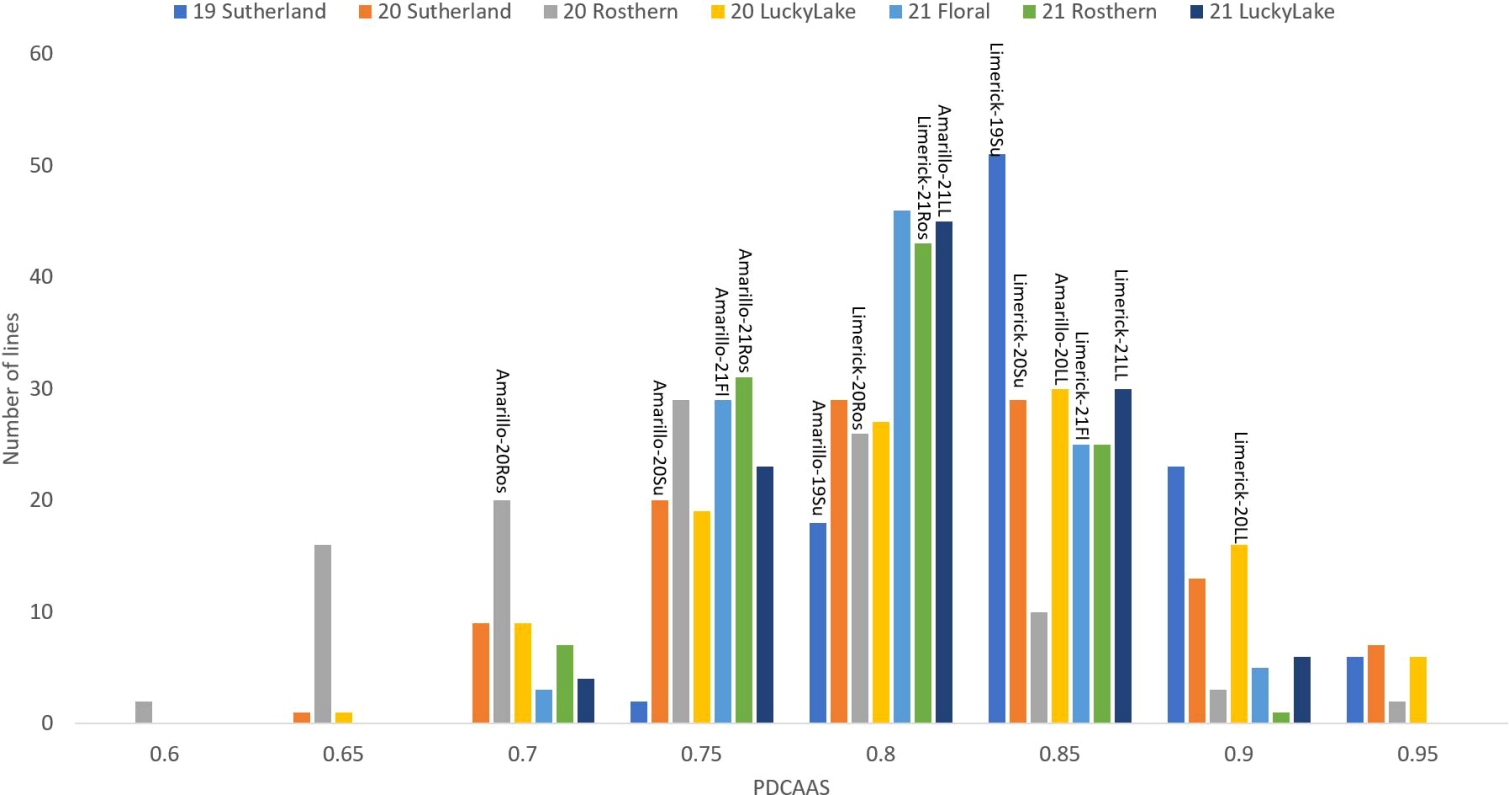
Frequency distribution of PDCAAS of PR-25 across seven station-years based on the average of biological replicates for each line in each station-year (two biological replicates in 2019 Sutherland and three biological replicates for the rest of the station-years), and the averaged PDCAAS for the parents of PR-25 in each station-year.

Two sample t-tests with equal variance were conducted on protein related traits, including the concentration of protein and each of 18 amino acids, for the parents of PR-25, CDC Amarillo and CDC Limerick (**Table 2**). Data of CDC Amarillo and CDC Limerick from all 7 station-years were used for the t-test. A Pearson correlation analysis was performed among protein quality traits, including total protein, protein digestibility (*in vitro*), PDCAAS and 18 amino acids found in pea seeds (**Table 3**), based on the average of 7 station-years data (2 biological replicates in 2019 Sutherland, 3 biological replicates in 2020 Sutherland, 2020 Rosthern, 2020 Lucky Lake, 2021 Floral, 2021 Rosthern, 2021 Lucky Lake) for each trait. Analysis of variance (ANOVA) was conducted for amino acids of interest, including methionine, cystine, tryptophan and lysine using 7 station-years data; ANOVA of multi-environment function was conducted in QTL IciMapping software (**Table 4**). A comparison of amino acid profiles was made among years 2019, 2020, 2021 and the average of all 7 station-years (**Figure 1**). A comparison of amino acid profiles was also made between CDC Amarillo and CDC Limerick based on their average of 7 station-years (**Figure 2**). Each amino acid profile represented the average percentage of each amino acid in pea seeds in the given year. Frequency distribution of methionine + cysteine concentration, tryptophan concentration, lysine concentration, protein digestibility (*in vitro*) and PDCAAS was made based on the average of biological replicates for each line in the given year-location (**Figures 3**–**7**).

**Table 2:** Two sample t-tests with equal variance between the parents of PR-25, CDC Amarillo and CDC Limerick, across 7 station-years.

AA	Ala	Met	Cys	His	Ser	Arg	Gly
**p value**	0.188ns	<0.001***	0.002**	<0.001***	0.001**	0.031*	0.036*
AA	Asp	Glu	Thr	Pro	Lys	Tyr	Val
**p value**	0.03*	<0.001***	0.039*	0.001**	<0.001***	0.004**	0.007**
AA	Ile	Leu	Phe	Tryp	Protein		
**p value**	0.010*	0.002**	0.010*	0.021*	0.004**		

Significance levels for the correlation coefficient (r) is denoted by the symbols *, **, ***, for P< 0.05, P< 0.01, P< 0.001 or not significant (ns), respectively.

**Table 3:** Correlation analysis among total protein, protein digestibility (*in vitro*), PDCAAS and 18 amino acids found in pea seed.

	Ala	Met	Cys	His	Ser	Arg	Gly	Asp	Glu	Thr	Pro	Lys	Tyr	Val	Ile	Leu	Phe	Trp	Protein	IVPD
**Met**	0.52***																			
**Cys**	0.25**	0.51***																		
**His**	0.64***	0.37***	0.18ns																	
**Ser**	0.52***	0.11ns	0.27**	0.73***																
**Arg**	0.76***	0.63***	0.51***	0.56***	0.56***															
**Gly**	0.80***	0.14ns	0.16ns	0.75***	0.82***	0.57***														
**Asp**	0.89***	0.51***	0.42***	0.70***	0.70***	0.87***	0.78***													
**Glu**	0.76***	0.37***	0.34***	0.86***	0.89***	0.72***	0.88***	0.89***												
**Thr**	0.84***	0.23*	0.15ns	0.77***	0.85***	0.64***	0.96***	0.84***	0.90***											
**Pro**	0.75***	0.24*	0.35***	0.78***	0.88***	0.74***	0.92***	0.89***	0.94***	0.91***										
**Lys**	0.68***	0.13ns	0.15ns	0.84***	0.86***	0.58***	0.89***	0.80***	0.91***	0.90***	0.93***									
**Tyr**	0.50***	0.21*	0.38***	0.66***	0.83***	0.65***	0.69***	0.74***	0.81***	0.74***	0.83***	0.76***								
**Val**	0.76***	0.21*	0.24*	0.79***	0.90***	0.70***	0.94***	0.87***	0.94***	0.96***	0.98***	0.94***	0.84***							
**Ile**	0.75***	0.24*	0.28**	0.75***	0.88***	0.73***	0.91***	0.90***	0.94***	0.92***	0.98***	0.91***	0.84***	0.98***						
**Leu**	0.79***	0.31**	0.30**	0.78***	0.89***	0.76***	0.90***	0.92***	0.97***	0.93***	0.97***	0.92***	0.83***	0.98***	0.99***					
**Phe**	0.49***	-0.02ns	0.17ns	0.73***	0.94***	0.51***	0.83***	0.70***	0.87***	0.83***	0.91***	0.92***	0.85***	0.92***	0.91***	0.90***				
**Trp**	0.73***	0.73***	0.52***	0.66***	0.58***	0.81***	0.6***	0.79***	0.74***	0.68***	0.68***	0.55***	0.68***	0.66***	0.68***	0.71***	0.46***			
**Protein**	0.74***	0.47***	0.49***	0.80***	0.83***	0.85***	0.78***	0.92***	0.94***	0.83***	0.92***	0.84***	0.84***	0.90***	0.91***	0.93***	0.81***	0.81***		
**IVPD**	0.12ns	0.22ns	0.11ns	0.07ns	-0.04ns	0.1ns	-0.02ns	0.13ns	0.05ns	0.04ns	0.03ns	0.04ns	0.06ns	0.04ns	0.05ns	0.06ns	-0.01ns	0.13ns	0.10ns	
**PDCAAS**	-0.09ns	0.39***	0.23*	-0.21ns	-0.36***	-0.09ns	-0.33***	-0.13ns	-0.26**	-0.29**	-0.29**	-0.33**	-0.23*	-0.30**	-0.28**	-0.28**	-0.38***	0.00ns	-0.19*	0.88***

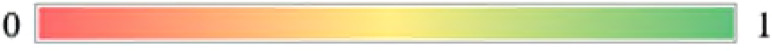

Significance levels for the correlation coefficient (r) is denoted by the symbols *, **, ***, for P < 0.05, P < 0.01, P < 0.001 or not significant (ns), respectively.

**Table 4:** ANOVA analysis on methionine, cysteine, tryptophan and lysine.

Methionine						
Source of Variation	DF	SS	MS	F	P-value	H^2^ (%)
Block	13	0.0055	0.0004	6.2120	<0.01***	25.5%
Genotype	110	0.0858	0.0008	11.5416	<0.01***	
Station-year	6	1.4502	0.2417	3575.5540	<0.01***	
GE_Interaction	651	0.1154	0.0002	2.6215	<0.01***	
Model	780	1.6568	0.0021	31.4234	<0.01***	
Error	1385	0.0936	0.0001			
Total	2165	1.7504				
Cysteine						
Source of Variation	DF	SS	MS	F	P-value	H^2^ (%)
Block	13	0.17	0.01	40.95	<0.01***	16.6%
Genotype	110	0.21	0.00	6.00	<0.01***	
Station-year	6	1.26	0.21	648.36	<0.01***	
GE_Interaction	651	0.38	0.00	1.80	<0.01***	
Model	780	2.03	0.00	8.02	<0.01***	
Error	1385	0.45	0.00			
Total	2165	2.47				
Tryptophan						
Source of Variation	DF	SS	MS	F	P-value	H^2^ (%)
Block	13	0.0047	0.0004	7.3664	<0.01***	26.6%
Genotype	110	0.0507	0.0005	9.4113	<0.01***	
Station-year	6	1.9084	0.3181	6497.8110	<0.01***	
GE_Interaction	651	0.0488	0.0001	1.5312	<0.01***	
Model	780	2.0126	0.0026	52.7111	<0.01***	
Error	1385	0.0678	0.0000			
Total	2165	2.0804				
Lysine						
Source of Variation	DF	SS	MS	F	P-value	H^2^ (%)
Block	13	0.58	0.04	11.13	<0.01***	15.8%
Genotype	110	4.00	0.04	9.13	<0.01***	
Station-year	6	12.41	2.07	519.56	<0.01***	
GE_Interaction	651	11.45	0.02	4.42	<0.01***	
Model	780	28.44	0.04	9.16	<0.01***	
Error	1385	5.52	0.00			
Total	2165	33.96				

Significance levels is denoted by the symbols ***, for P< 0.001. GE_Interaction was referred to gene-environment interactions. H^2^ is the heritability of the assessed traits.

### Genotyping, linkage map construction and QTL identification

PR-25 population was genotyped using Axiom^®^ 90K SNP array and a linkage map representing 901 unique loci and measuring 855.4 cM was reported by Zhou et al. (2022). This linkage map was used for QTL analysis in the current study. The nomenclature of the markers was in accordance with Axiom^®^ 90K SNP array. To increase the accuracy of QTL identification, QTL analyses were first performed individually in each station-year. Data of some station-years, in which their individual analysis didn’t include any significant QTL, were excluded and the rest were averaged and used for combined QTL analysis. Phenotypic traits, including methionine + cysteine concentration, tryptophan concentration, lysine concentration and *in vitro* protein digestibility, were assessed. QTL analysis was conducted *via* Windows QTL Cartographer (Wang et al., 2012), where cross type was set as Ri1, map function was set as Kosambi, analysis type was set as composite interval mapping, permutation time was set as 1000, significance level was set as 0.05, and walk speed was set as 1.0 Cm. Other detailed information of the QTL identification approach was described in Zhou et al., 2022.

## Results

The results of two sample t-test between the parents of PR-25 population, CDC Amarillo and CDC Limerick, showed that they differed significantly in their concentration of seed protein and most of the amino acids, except for alanine (**Table 2**). The average proportion of each amino acid from field trials conducted over three seasons is summarized in **Figure 1**. The average of each amino acid content from the 7 station-years can be ranked from top to bottom as: Glutamine (18.3%), aspartic acid (12.4%), lysine (7.7%), leucine (7.5%), arginine (7.5%), phenylalanine (5.2%), serine (5.1%), valine (4.9%), glycine (4.5%), isoleucine (4.4%), proline (4.4%), alanine (4.3%), threonine (3.9%), tyrosine (3.3%), histidine (2.9%), cysteine (1.4%), methionine (1.2%), tryptophan (1.1%). The average proportion of each amino acid of CDC Amarillo and CDC Limerick from 7 station-years is summarized in **Figure 2**. There were positive correlations among most amino acids (**Table 3**). Variation in amino acid profile was detected across the station-years. Analysis of variance of methionine, cysteine, tryptophan, and lysine showed that the phenotypic variation can be attributed to the effects of genotype, station-year, and the interaction of genotype and station-year (**Table 4**). Frequency distribution of methionine + cysteine concentration, tryptophan concentration, lysine concentration, protein digestibility and PDCAAS presented the ranges, the distribution of phenotypic scores of PR-25 in each station-year (**Figures 3**–**7**).

**Figure 8:**
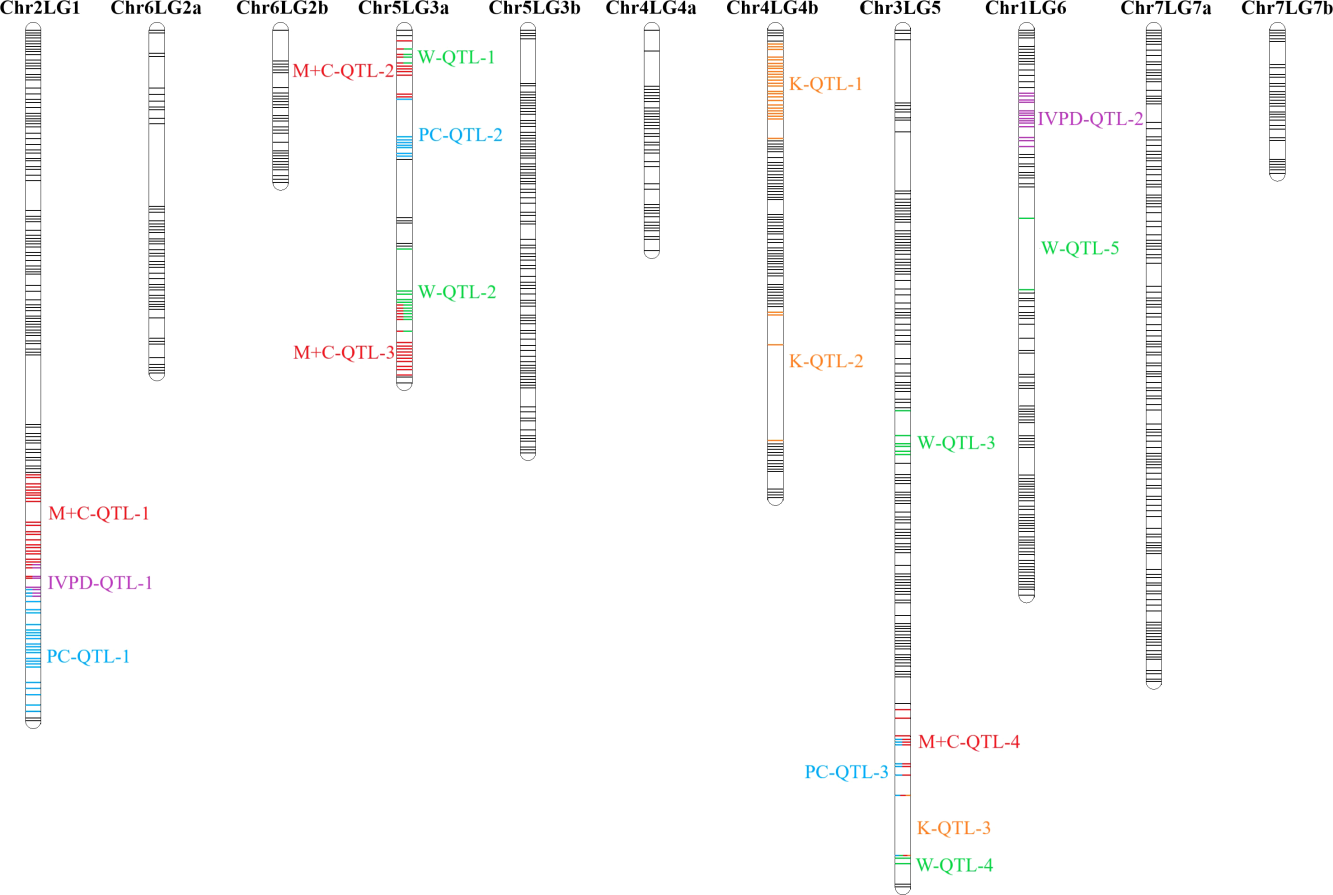
Combined QTL analysis in PR-25 reveals the QTLs associated for protein concentration (PC) (Zhou et al., 2022), *in vitro* protein digestibility (IVPD), lysine concentration (K), tryptophan concentration (W) and methionine + cysteine concentration (M+C). Combined analysis was based on averaged phenotypic data from station-years in which the QTL was significant (minimum of 3 of 7 station-years).

As the primary interest of this research was related to both the abundant and limiting amino acids, hence, lysine, as the abundant amino acid, and tryptophan, cysteine and methionine, as the limiting amino acids, were selected for QTL analysis (**Table 4**). Cysteine and methionine were combined in QTL analysis since they belong to same metabolic pathway (Ravanel et al, 1998). ANOVA showed that environment and genotype × environment interactions had significant contributions to the variation in amino acid concentrations. Therefore, QTL analysis was based on averaged data from selected station-years from which their individual analysis showed significant QTLs. A total of 14 QTLs were identified associated with protein quality traits of interest and they were significant in at least 4 of 7 station-years when compared individually. Four QTLs were identified associated with the methionine + cysteine concentration. Met+Cys-QTL-1 was found on chromosome 2 and it explained 15% of the phenotypic variation, its flanking markers were Chr2LG1_244771437/Chr2LG1_287501555 and there were 22 markers in between; Met+Cys-QTL-2 was found on chromosome 5 and it explained 11% of the phenotypic variation, its flanking markers were Chr5LG3_4173823/Chr5LG3_15801800 and there were 10 markers in between; Met+Cys-QTL-3 was also found on chromosome 5 and it explained 16% of the phenotypic variation, its flanking markers were Chr5LG3_101924498/Chr5LG3_137457380 and there were 10 markers in between; Met+Cys-QTL-4 was found on chromosome 3 and it explained 10% of the phenotypic variation, its flanking markers were Chr3LG5_120117355/Chr3LG5_408080154 and there were 8 markers in between (**Figure 8**). Chr2LG1_259006623, Chr5LG3_5113345, Chr5LG3_137457380 were the loci within the peak region of each of these three methionine + cysteine associated QTLs. Five QTLs were identified associated with tryptophan concentration, Trp-QTL-1 was found on chromosome 5 and it explained 8% of the phenotypic variation, its flanking markers were Chr5LG3_5127342/Chr5LG3_7509381 and there were 2 markers in between; Trp-QTL-2 was also found on chromosome 5 and it explained 13% of the phenotypic variation, its flanking markers were Chr5LG3_67663653/Chr5LG3_112710798 and there were 10 markers in between; Trp-QTL-3 was found on chromosome 3 and it explained 9% of the phenotypic variation, its flanking markers were Chr3LG5_185794949/Chr3LG5_198663551 and there were 4 markers in between; Trp-QTL-4 was also found on chromosome 3 and it explained 8% of the phenotypic variation, its flanking markers were Chr3LG5_424086163/Chr3LG5_455814220 and there were 2 markers in between; Trp-QTL-5 was found on chromosome 1 and it explained 9% of the phenotypic variation, its flanking markers were Chr1LG6_132953926/Chr1LG6_233117537 and there were 2 markers in between. Chr5LG3_5381756, Chr5LG3_84309239, Chr3LG5_197202364, Chr1LG6_233117537 were the loci within the peak region of each of these four tryptophan associated QTLs. Three QTLs were identified associated with lysine concentration. Lys-QTL-1 was found on chromosome 4 and it explained 21% of the phenotypic variation, its flanking markers were Chr4LG4_185310109/Chr4LG4_218381712 and there were 23 markers in between; Lys-QTL-2 was also found on chromosome 4 and it explained 15% of the phenotypic variation, its flanking markers were Sc02659_148875/Chr4LG4_417303831 and there were 2 markers in between; Lys-QTL-3 was found on chromosome 1 and it explained 10% of the phenotypic variation, its flanking markers were Chr3LG5_424086163/Chr3LG5_437233435 and there were 2 markers in between. Chr4LG4_206035753, Chr4LG4_326486541, Chr3LG5_437233435 were the loci within the peak region of each of these three lysine associated QTLs. Two QTLs were identified associated with IVPD. IVPD-QTL-1 was found on chromosome 2 and it explained 10% of the phenotypic variation, its flanking markers were Chr2LG1_285985643/Chr2LG1_290867919 and there were 6 markers in between; IVPD-QTL-2 was found on chromosome 4 and it explained 11% of the phenotypic variation, its flanking markers were Chr1LG6_36689547/Chr1LG6_71617678 and there were 12 markers in between. Chr2LG1_291265214, Chr1LG6_41413580 were the loci within the peak region of each of these two *in vitro* protein digestibility associated QTLs. In addition, a set of QTL analyses were conducted in each station-year for methionine + cysteine concentration, tryptophan concentration, lysine concentration and protein digestibility, as shown in **Table 5**. The positions of identified QTLs, based on the individual QTL analysis from each station-year, were presented in **Supplementary Figures 1**–**4**.

**Table 5:** Detected quantitative trait loci (QTL) for methionine + cysteine, tryptophan, lysine concentration, and *in vitro* protein digestibility in PR-25 using composite interval mapping (CIM).

Methionine + Cysteine - QTLs							
Name of QTL	Chromosome/Linkage group	Station-year	Position/Peak (cM)	Flanking markers	LOD score	R^2^ (%)	Additive effect
Met+Cys-QTL-1	Chr2LG1	Combined	79.3-97.3/86.6	Chr2LG1_244771437/ Chr2LG1_287501555	6.4	15	0.006
		20Rosthern	87.7-101.2/89.0	Chr2LG1_257899664/Chr2LG1_457180999	3.6	9	0.008
		20LuckyLake	88.3-94.8/89.6	Chr2LG1_259390540/ Chr2LG1_285985643	3.5	11	0.008
		21Floral	84.4-96.1/87.0	Chr2LG1_259006623/Chr2LG1_287178718	3.8	10	0.006
		21LuckyLake	82.5-103.1/94.0	Chr2LG1_252806647/Chr2LG1_293686819	7.5	18	0.009
Met+Cys-QTL-2	Chr5LG3a	Combined	1.8-11.6/4.5	Chr5LG3_4173823/Chr5LG3_15801800	4.1	11	-0.005
		20Sutherland	16.8-31.6/26.4	Chr5LG3_23306250/ Chr5LG3_47058351	4.2	14	-0.01
		21Floral	1.0-12.2/5.3	Chr5LG3_1308458/Chr5LG3_15801800	3.8	11	-0.007
		21LuckyLake	1.5-5.9/3.3	Chr5LG3_4173823/Chr5LG3_7509381	3.2	7	-0.005
Met+Cys-QTL-3	Chr5LG3a	Combined	48.4-60.9/59.1	Chr5LG3_101924498/Chr5LG3_137457380	7.3	16	-0.006
		20Rosthern	46.9-61.1/56.2	Chr5LG3_83086224/Chr5LG3_146553297	4.4	12	-0.008
		20LuckyLake	57.2-61.3/57.6	Chr5LG3_137457380/Chr5LG3_146553297	3.5	11	-0.009
		21LuckyLake	48.4-61.1/61.0	Chr5LG3_101924498/Chr5LG3_146553297	6.9	17	-0.008
		21Floral	56.7-57.6/57.2	Chr5LG3_131198978/Chr5LG3_137724142	3.1	8	-0.005
Met+Cys-QTL-4	Chr3LG5	Combined	118.3-142.1/130.8	Chr3LG5_120117355/Chr3LG5_408080154	4.4	10	-0.005
		20Sutherland	113.7-129.4/124.3	Chr3LG5_236004028/Chr3LG5_422388773	3.5	9	-0.006
		20Rosthern	114.9-143.1/120.4	Chr3LG5_236004028/ Chr3LG5_437233435	7.1	20	-0.01
		21LuckyLake	135.3-149.5/141.5	Chr3LG5_424086163/ Chr3LG5_459895536	4.8	12	-0.007
Tryptophan – QTLs							
Name of QTL	Chromosome/Linkage group	Station-year	Position/Peak (cM)	Flanking markers	LOD score	R^2^ (%)	Additive effect
Trp-QTL-1	Chr5LG3a	Combined	2.0-10.9/4.5	Chr5LG3_5127342/Chr5LG3_7509381	4.9	8	-0.001
		20Sutherland	5.2-11.6/9.9	Chr5LG3_7509381/Chr5LG3_17377451	4.6	13	-0.002
		20Rosthern	3.2-11.0/4.3	Chr5LG3_5127342/Chr5LG3_17355584	4.3	14	-0.002
		21LuckyLake	1.9-19.3/4.3	Chr5LG3_4173823/Chr5LG3_23359112	5.5	12	-0.003
Trp-QTL-2	Chr5LG3a	Combined	56.5-60.9/59.1	Chr5LG3_67663653/Chr5LG3_112710798	4.3	13	-0.001
		20Rosthern	47.7-60.5/57.4	Chr5LG3_459165288/ Chr5LG3_146553297	4.7	11	-0.002
		20LuckyLake	56.7-61.0/59.3	Chr5LG3_135166280/ Chr5LG3_146553297	3.4	8	-0.001
		21Floral	43.8-57.4/47.1	Chr5LG3_84309239/Chr5LG3_137724142	4.5	11	-0.003
		21LuckyLake	52.2-61.0/57.5	Chr5LG3_112710798/Chr5LG3_146553297	5.4	12	-0.003
Trp-QTL-3	Chr3LG5	Combined	62.5-75.4/65.1	Chr3LG5_185794949/Chr3LG5_198663551	3.8	9	-0.001
		20Sutherland	56.1-72.8/64.5	Chr3LG5_152019637/ Chr3LG5_197202364	4.8	11	-0.002
		20LuckyLake	62.5-74.8/66.9	Chr3LG5_163108809/Chr3LG5_198663551	4.4	12	-0.002
		21LuckyLake	62.5-68.3/63.8	Chr3LG5_163108809/Chr3LG5_185794949	3.8	8	-0.002
Trp-QTL-4	Chr3LG5	Combined	135.4-143.2/139.7	Chr3LG5_424086163/Chr3LG5_455814220	3.4	8	-0.015
		20Sutherland	126.3-145.7/138.5	Chr3LG5_13646657/ Chr3LG5_455814220	4.6	13	-0.002
		20Rosthern	136.0-149.5/148.4	Chr3LG5_424086163/Chr3LG5_459895536	3.7	9	-0.002
		20LuckyLake	118.4-124.6/120.5	Chr3LG5_120117355/Chr3LG5_24108451	3.1	9	-0.002
		21LuckyLake	136.0-148.5/140.5	Chr3LG5_424086163/ Chr3LG5_455088720	4.3	11	-0.003
Trp-QTL-5	Chr1LG6	Combined	34.6-50.4/42.1	Chr1LG6_132953926/Chr1LG6_233117537	4.6	9	0.001
		20Rosthern	27.0-47.2/43.5	Chr1LG6_97211857/Chr1LG6_240693356	4.1	10	0.003
		20LuckyLake	45.1-49.6/47.1	Chr1LG6_233117537/Chr1LG6_261967742	3.4	8	0.002
		21Floral	41.3-61.9/48.6	Chr1LG6_233117537/Chr1LG6_305454704	4.2	10	0.003
Lysine – QTLs							
Name of QTL	Chromosome/Linkage group	Station-year	Position/Peak (cM)	Flanking markers	LOD score	R^2^ (%)	Additive effect
Lys-QTL-1	Chr4LG4b	Combined	3.6-18.9/8.3	Chr4LG4_185310109/Chr4LG4_218381712	6.7	21	-0.02
		20Sutherland	7.8-13.2/10.8	Chr4LG4_194957049/Chr4LG4_207119930	5.3	17	-0.01
		20LuckyLake	11.8-21.4/13.1	Chr4LG4_203961784/Chr4LG4_226603806	4.9	14	-0.01
		21Floral	2.4-19.0/8.3	Chr4LG4_185310109/Chr1LG6_334873830	7.7	20	-0.03
Lys-QTL-2	Chr4LG4b	Combined	52.3-60.8/55.5	Sc02659_148875/Chr4LG4_417303831	3.5	15	0.02
		20Rosthern	45.15-55.3/50.4	Chr4LG4_316816169/Chr4LG4_326486541	3.2	13	0.01
		21Floral	50.0-73.7/58.6	Chr4LG4_438891008/Chr4LG4_418348946	3.7	16	0.03
		21LuckyLake	55.3-76.2/60.2	Chr4LG4_326486541/Chr4LG4_438079536	3.1	12	0.01
Lys-QTL-3	Chr3LG5	Combined	134.2-144.8/141.6	Chr3LG5_424086163/Chr3LG5_437233435	4.3	10	-0.01
		20LuckyLake	107.3-118.4/113.2	Chr3LG5_257364623/Chr3LG5_120117355	3.8	8	-0.01
		21Floral	125.6-150.1/140.2	Chr3LG5_13646657/Chr3LG5_459895536	4.1	9	-0.02
		21LuckyLake	124.1-146.5/138.9	Chr1LG6_347327176/Chr3LG5_455088720	4.1	10	-0.02
Protein Digestibility (*in vitro*)- QTLs							
Name of QTL	Chromosome/Linkage group	Station-year	Position/Peak (cM)	Flanking markers	LOD score	R^2^ (%)	Additive effect
IVPD-QTL-1	Chr2LG1	Combined	95.5-100.5/98.4	Chr2LG1_285985643/Chr2LG1_290867919	3.3	10	1.27
		21Floral	93.5-103.8/98.6	Chr2LG1_271201976/Chr2LG1_295431002	4.1	13	1.55
		21Rosthern	92.0-101.7/94.5	Chr2LG1_267076305/Chr2LG1_457180999	3.3	10	1.26
		21LuckyLake	94.0-98.6/94.5	Chr2LG1_278779827/Chr2LG1_291265214	3.1	10	1.196
IVPD-QTL-2	Chr1LG6	Combined	10.9-20.5/14.4	Chr1LG6_36689547/Chr1LG6_71617678	3.5	11	-1.12
		19Sutherland	0.2-1.9/0.9	Chr1LG6_24608817/Chr1LG6_27085223	3.1	9	-1.03
		20Sutherland	9.7-23.8/15.5	Chr1LG6_33266075/Chr1LG6_84517828	4.3	14	-1.34
		20Rosthern	9.0-24.0/14.4	Chr1LG6_30581935/Chr1LG6_87699075	3.3	10	-1.00
		21Rosthern	11.0-21.3/14.6	Chr1LG6_34300922/Chr1LG6_81358908	3.4	10	-1.27
		21LuckyLake	9.7-21.9/10.9	Chr1LG6_33266075/Chr1LG6_81358908	3.8	12	-1.33

Combined analysis was based on averaged phenotypic data from station-years in which the QTL was significant. Individual QTL analysis was based on averaged phenotypic data of biological replicates in each station-year. Additive effects were calculated as the average performance of lines carrying A allele from CDC Amarillo minus the average performance of lines carrying B allele from CDC Limerick.

## Discussion

Development of pea varieties with high seed protein concentration and quality is necessary to fulfill the growing plant-based protein demand. To accomplish this goal, a better understanding of the underlying genetic control of the protein related traits is required.

Pea recombinant inbred line population PR-25, derived from the cross of CDC Amarillo and CDC Limerick, was developed specifically for the study of protein related traits. The variation in concentration of individual amino acids between CDC Amarillo and CDC Limerick ranged from 10 to 27%. They were significantly different in the concentration of almost all amino acids, except for alanine. These ensured sufficient diversity within PR-25 population to identify amino acid related QTLs. CDC Amarillo and CDC Limerick are widely grown in western Canada for their good yield and protein concentration, therefore, PR-25 is an ideal population for the research of protein-quality traits and could potentially avoid tradeoff between favorable traits.

HPLC analysis of individual amino acids is a destructive method which requires protein digestion steps. However, the unique attributes of some amino acids increase the complexity of the digestion steps. In comparison to HPLC analysis, NIR analysis is a non-destructive, high throughput and cost-efficient method to assess amino acid profile for pea protein. Amino acid profile of a thousand samples could be assessed in a week by using NIR analysis, while it may take months if using HPLC analysis. In the current study, the calibration formula developed based on HPLC quantification of amino acids in pea genotypes was used for NIR based prediction of amino acids. For most amino acids, their correlation coefficient values (r) were above 0.9. For the limiting amino acids, such as methionine, cystine and tryptophan, their r values were also acceptable, which were 0.733, 0.833 and 0.855, respectively.

Pea protein is limited in sulfur amino acids and is abundant in lysine, thus complements cereal protein to provide complete plant protein. There is a debate on whether the improvements of limiting amino acids in certain crops are necessary since the other option would always be paired up with a different crop to provide complete nutrition. Though it seems less cost-effective to enhance the nutritional attributes of a single crop, this approach has several benefits. When introducing more ingredients into a food product, there is an increased risk of increasing the allergenicity of the product. Secondly, having more ingredients in a food product often means more food processing steps, including masking of unpleasant flavors, or more addition of food coloring which increases the cost. Meanwhile, over-processing is an issue that causes a decline in consumer acceptance. There is increased willingness among consumers to opt for products with fewer ingredients, driven by their consciousness for more healthy diets. Furthermore, climate change has different impacts on crops, and some are more severely influenced (Raza et al., 2019). The improvement of protein quality in single crop would contribute to food security and provide strength for agricultural businesses.

In PR-25, the averaged *in vitro* PDCAAS of 7 station-years ranged from 0.73 to 0.94. The PDCAAS of pea protein isolates was reported as 0.86 for children and 0.93 for adults by the FDA (2019). Some lines in PR-25 had lower PDCAAS than what was reported by the FDA. However, the measurements were conducted on whole pea seeds in the current study, while FDA measured PDCAAS on protein isolates. Since the fractionation process had positive effects on PDCAAS *via* improving the protein digestibility, whole seed samples were expected to have a lower PDCAAS than protein concentrates or isolates (Rivera Del Rio et al., 2022).

Some variation in amino acid profile was detected among station-years. These variations can be attributed to the effects of environments, and the interaction between genotype and environment. These abiotic factors impact individual amino acids differently and some amino acids, for instance, arginine and phenylalanine, had larger variations across station-years compared to others, which would lead to a change of their proportion in pea protein. However, these proportional changes in some amino acids only had limited impact on the overall amino acid profile as the profile didn’t change significantly across station-years.

All 18 amino acids assessed in this study were found positively correlated with total protein concentration, in most cases with correlation coefficients above 0.8. Meanwhile, close proximities or overlaps were found on several protein-related traits. Met+Cys-QTL-1 was found adjacent to PC-QTL-1 on chromosome 2, where IVPD-QTL-1 was located in between. Trp-QTL-1 was found within Met+Cys-QTL-2 on one end of chromosome 5 linkage group 3a and Trp-QTL-2 overlapped with Met+Cys-QTL-3 on another end. Met+Cys-QTL-4, PC-QTL-3, Lys-QTL-3 and Trp-QTL-4 were found overlapped on one end of Chromosome 3. Though overlaps were found among these protein-related traits, it didn’t necessarily lead to strong correlations. For instance, the correlation between protein concentration and methionine + cysteine concentration was 0.55, between IVPD and protein concentrations was 0.10, between IVPD and methionine + cysteine concentration was 0.17, between tryptophan concentration and methionine + cysteine concentration was 0.69. The main reason was that all protein associated traits assessed in this study are quantitative traits that are regulated by multiple loci. The identified QTL regions only explained small portions of the phenotypic variations and hence, though some of the QTL regions of these traits were in close proximity, their correlations were not very strong. Yet, within these overlapped QTL regions, there are potentials to find pleiotropic QTLs that control multiple protein quality traits of pea, as the research by Li et al. (2018) had identified several pleiotropic QTLs that associated with multiple amino acids concentrations in soybeans.

QTL analyses on pea seed traits including protein and mineral concentration, seed yield, thousand seed weight, seed number per plant, have been conducted by several research programs in the past two decades and numerous QTL had been identified associated with these traits (Tar'an et al., 2004; Ma et al., 2017; Dissanayaka et al., 2020; Klein et al., 2020; Zhou et al., 2022). Protein digestibility and amino acid profile have also been studied for deeper understanding of the functional attributes of pea protein (Nosworthy & House, 2017; Cabuk et al., 2018). However, none of the previous publications was related to QTL analysis for pea protein digestibility and amino acids concentration, and the QTLs identified in this study provide valuable information of the underlying genetic control of these traits. Twelve loci were found within the peak regions of the QTLs identified in this study, three were associated with methionine + cysteine concentration, four were associated with tryptophan concentration, three were associated with lysine concentration, and two were associated with *in vitro* protein digestibility. The information of these loci will be beneficial for developing markers to facilitate the selection of high protein-quality varieties in pea breeding. Several lines in PR-25, including PR-25-2, PR-25-46-PR-25-53, PR-25-86 and PR-25-96, had high concentrations of methionine, cysteine and tryptophan, as well as good protein concentration and grain yield. These lines had consistently good performance in protein quality traits across 7 station-years without compromise on other valued traits. These lines could be the potential high protein quality varieties or could be used as parental materials to develop varieties with better performance.

## Conclusion

The effect of genotype x environment interaction on the amino acid concentrations of pea cultivars is significant. The segregation pattern of amino acid concentrations in PR-25 population combined with the NIR-based predictions offers a possibility for high throughput selection of breeding lines for amino acid concentrations. Three QTLs were found associated with methionine + cysteine concentration, four QTLs were found associated with the tryptophan concentration, three QTLs were found associated with lysine concentration and two QTLs were found associated with *in vitro* protein digestibility. Overlaps were found among protein-related traits on chromosome 2 and chromosome 5. These identified QTL regions have a potential for use in marker-assisted selection of protein quality traits.

## Data availability statement

The raw data supporting the conclusions of this article will be made available by the authors, without undue reservation.

## Author contributions

TW, BT and KG conceptualized the study and secured funding for this study through a research grant from the Saskatchewan Ministry of Agriculture. KG developed the RIL population and conducted genotyping and linkage map construction. JZ conducted field trials, collected phenotypic data, conducted IVPD experiments, data curation, QTL analysis and wrote the manuscript. MN provided expert advice on IVPD experiments. JH designed methodology for amino acid profiling *via* NIR analysis. ZW conducted amino acid profiling. All authors contributed to the article and approved the submitted version.

## Supplementary material

Supplementary Figure 1 Individual QTL analysis from each station-year in PR-25 reveals the QTLs associated for methionine + cysteine concentration. Individual QTL analysis was based on average phenotypic data of biological replicates in each station-year.

Supplementary Figure 2 Individual QTL analysis from each station-year in PR-25 reveals the QTLs associated for tryptophan concentration. Individual QTL analysis was based on average phenotypic data of biological replicates in each station-year.

Supplementary Figure 3 Individual QTL analysis from each station-year in PR-25 reveals the QTLs associated for lysine concentration. Individual QTL analysis was based on average phenotypic data of biological replicates in each station-year.

Supplementary Figure 4 Individual QTL analysis from each station-year in PR-25 reveals the QTLs associated for *in vitro* protein digestibility. Individual QTL analysis was based on average phenotypic data of biological replicates in each station-year.
