# Supplementary figures and images for: Quantitative trait loci associated with amino acid concentration and in vitro protein digestibility in pea (Pisum sativum L.)
Source: Front Plant Sci. 2023 Mar 10;14:1083086. doi: 10.3389/fpls.2023.1083086 (PMC10038330; doi:10.3389/fpls.2023.1083086)

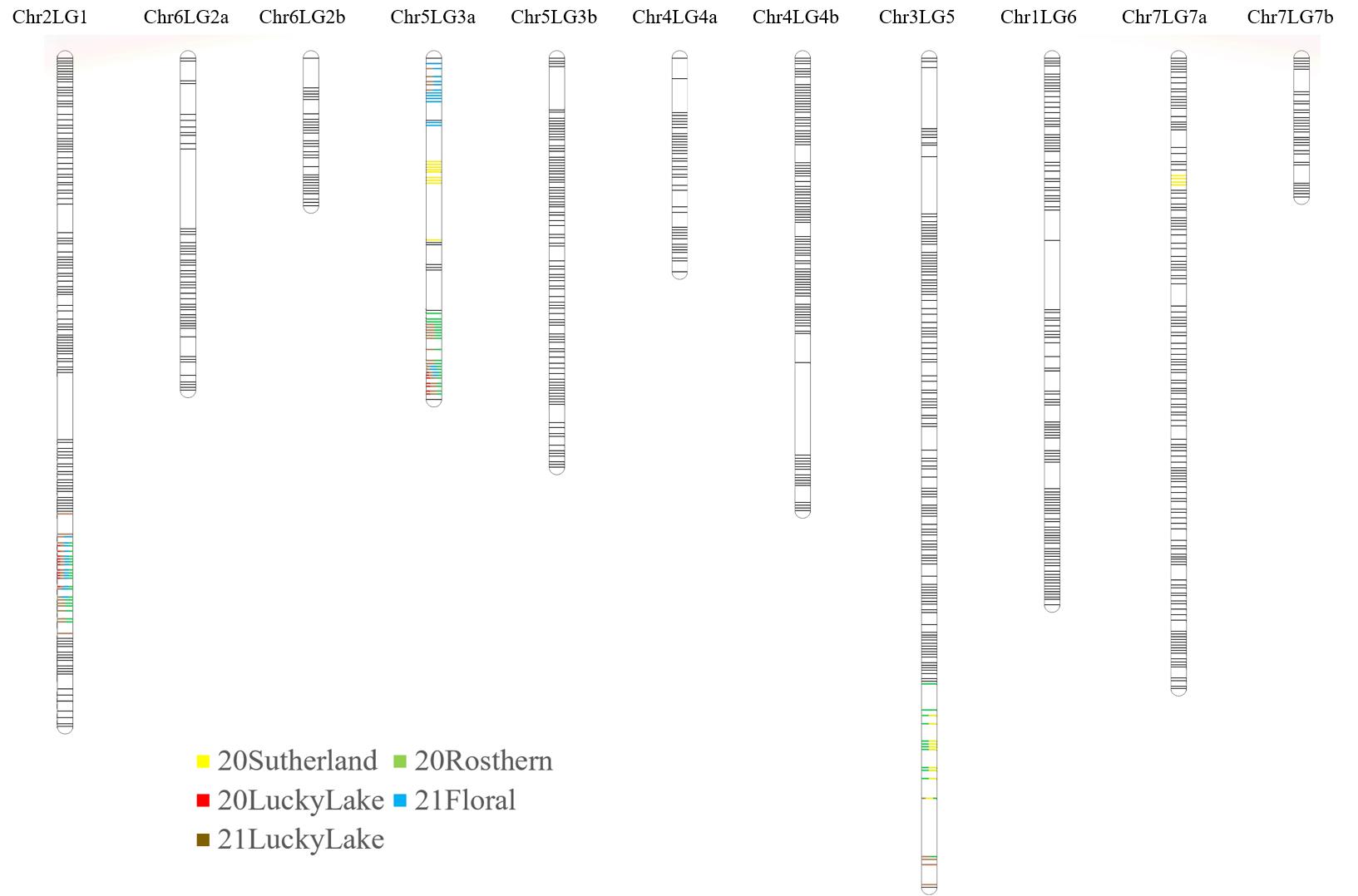

Supplement: Supplementary Figure 1 — Individual QTL analysis from each station-year in PR-25 reveals the QTLs associated for methionine + cysteine concentration. Individual QTL analysis was based on average phenotypic data of biological replicates in each station-year. [file Image_1.jpeg]

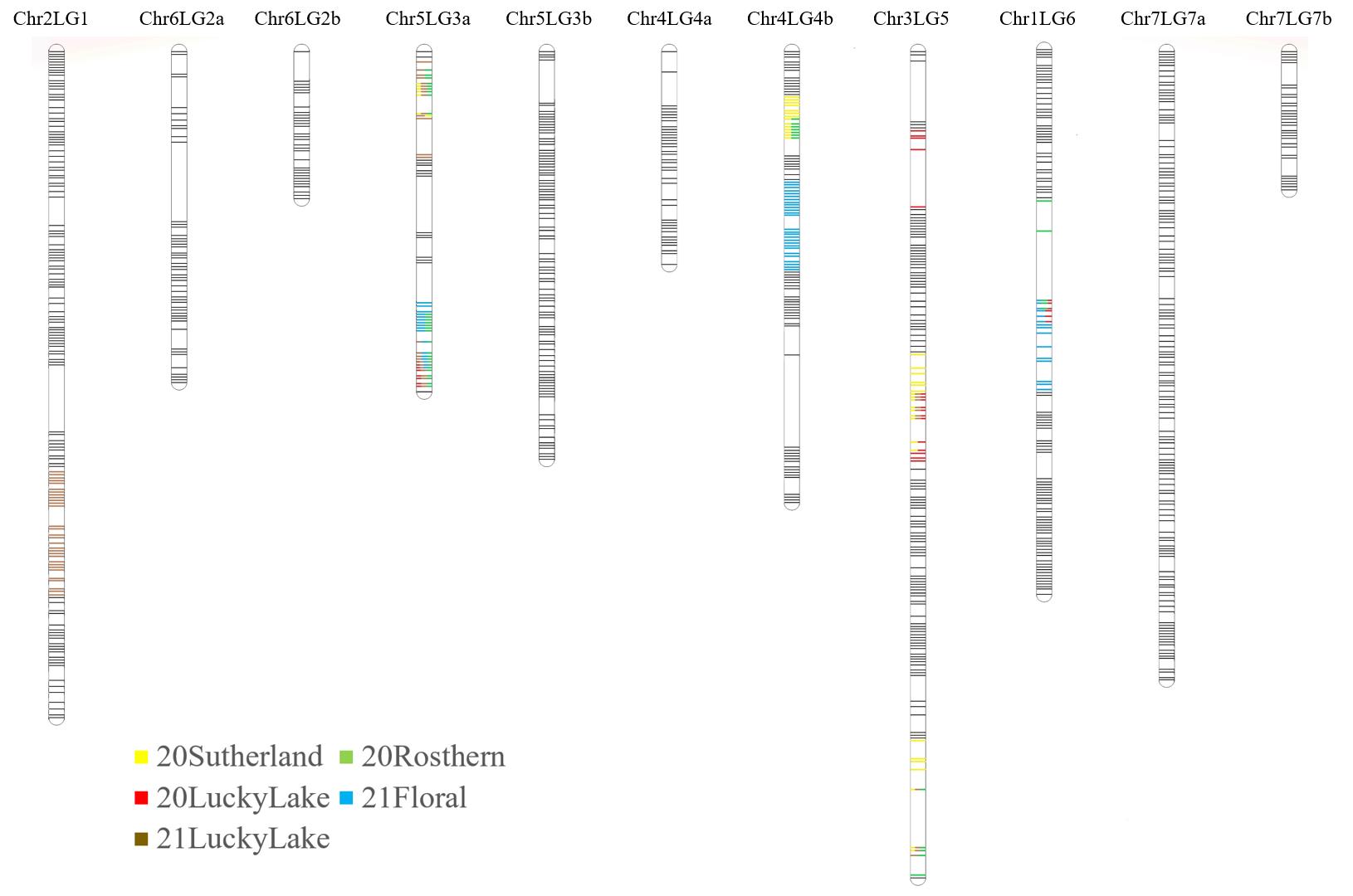

Supplement: Supplementary Figure 2 — Individual QTL analysis from each station-year in PR-25 reveals the QTLs associated for tryptophan concentration. Individual QTL analysis was based on average phenotypic data of biological replicates in each station-year. [file Image_2.jpeg]

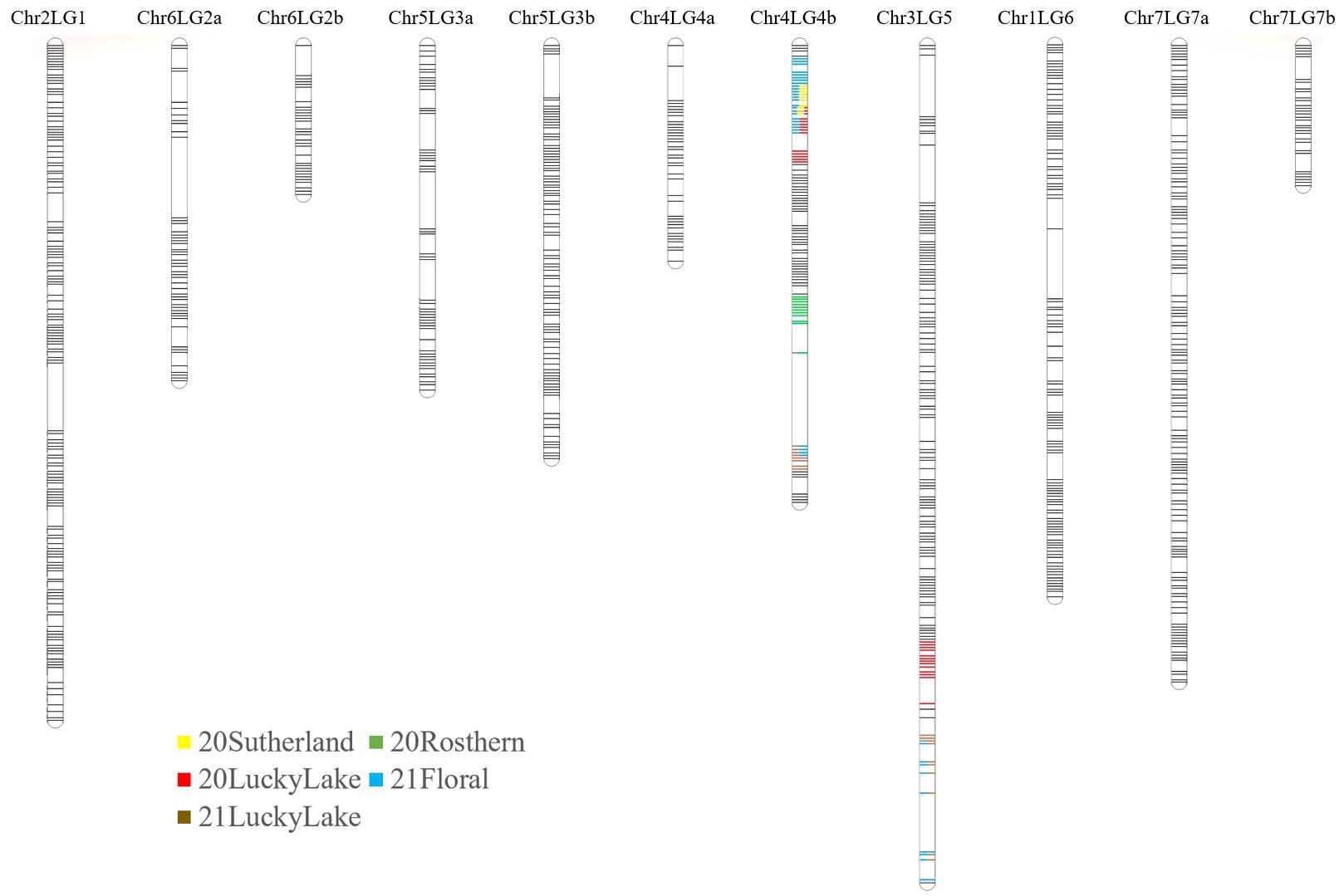

Supplement: Supplementary Figure 3 — Individual QTL analysis from each station-year in PR-25 reveals the QTLs associated for lysine concentration. Individual QTL analysis was based on average phenotypic data of biological replicates in each station-year. [file Image_3.jpeg]

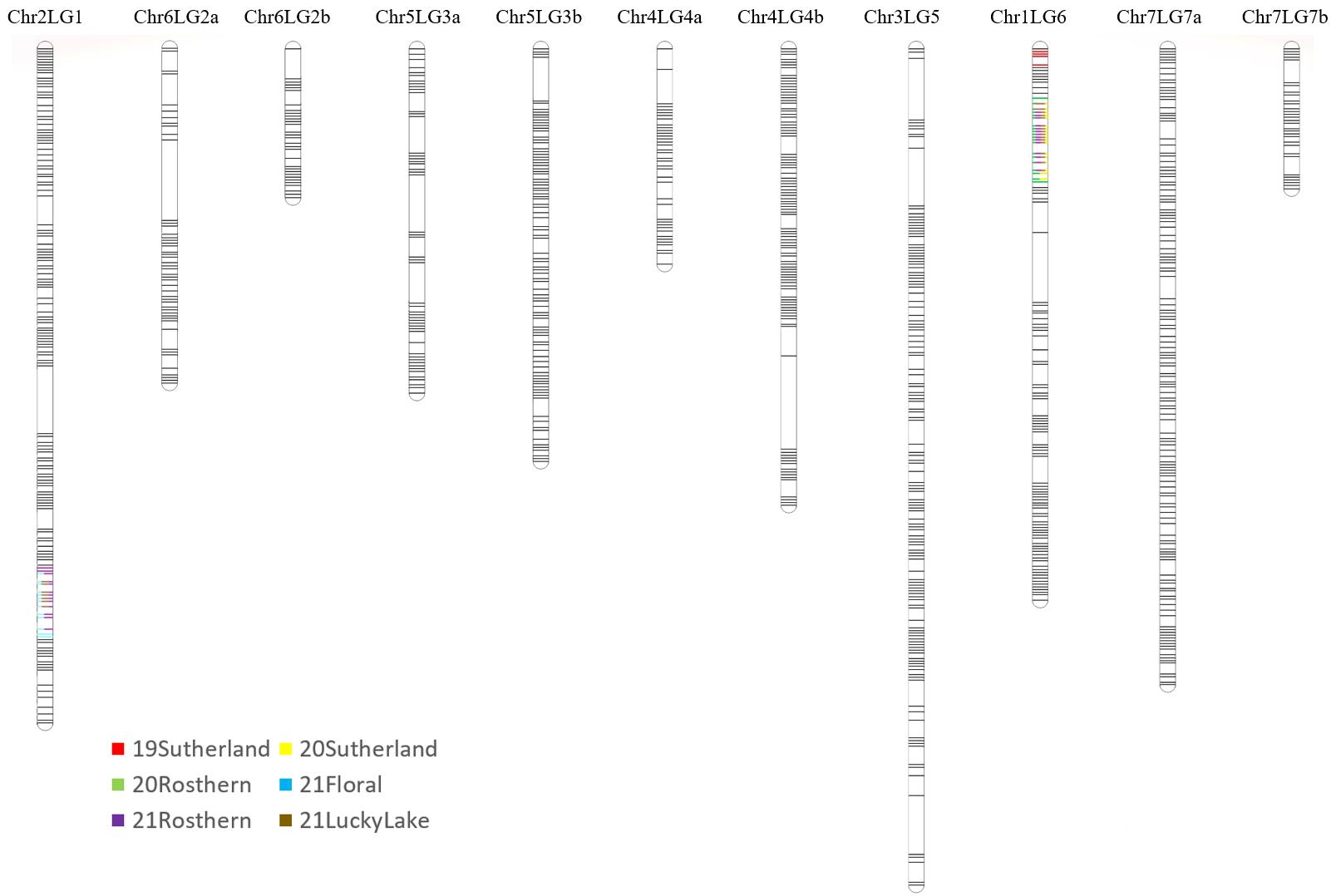

Supplement: Supplementary Figure 4 — Individual QTL analysis from each station-year in PR-25 reveals the QTLs associated for in vitro protein digestibility. Individual QTL analysis was based on average phenotypic data of biological replicates in each station-year. [file Image_4.jpeg]
